# Inhibition of Histone Deacetylases Facilitates Extinction and Attenuates Reinstatement of Nicotine Self-Administration in Rats

**DOI:** 10.1371/journal.pone.0124796

**Published:** 2015-04-16

**Authors:** Matthew R. Castino, Jennifer L. Cornish, Kelly J. Clemens

**Affiliations:** 1 Department of Psychology, Macquarie University, Sydney, NSW, Australia; 2 School of Psychology, The University of New South Wales, Sydney, NSW, Australia; University of Leicester, UNITED KINGDOM

## Abstract

Chromatin remodelling is integral to the formation of long-term memories. Recent evidence suggests that histone modification may play a role in the persistence of memories associated with drug use. The present series of experiments aimed to examine the effect of histone deacetylase (HDAC) inhibition on the extinction and reinstatement of nicotine self-administration. Rats were trained to intravenously self-administer nicotine for 12 days on a fixed-ratio 1 schedule. In Experiment 1, responding was then extinguished through removal of nicotine and response-contingent cues. After each extinction session, the HDAC inhibitor, sodium butyrate (NaB), was administered immediately, or six hours after each session. In Experiment 2, response-contingent cues remained available across extinction to increase rates of responding during this phase, and NaB was administered immediately after the session. Finally, in Experiment 3, the effect of NaB treatment on extinction of responding for sucrose pellets was assessed. Across all experiments reinstatement to the cue and/or the reward itself was then tested. In the first experiment, treatment with NaB significantly attenuated nicotine and nicotine + cue reinstatement when administered immediately, but not six hours after each extinction session. When administered after cue-extinction (Expt. 2), NaB treatment specifically facilitated the rate of extinction across sessions, indicating that HDAC inhibition enhanced consolidation of the extinction memory. In contrast, there was no effect of NaB on the extinction and reinstatement of sucrose-seeking (Expt. 3), indicating that the observed effects are specific to a drug context. These results provide the first demonstration that HDAC inhibition facilitates the extinction of responding for an intravenously self-administered drug of abuse and further highlight the potential of HDAC inhibitors in the treatment of drug addiction.

## Introduction

Tobacco addiction is a major cause of preventable ill health and death in western countries. Unfortunately, quitting is difficult, and among those that do quit, relapse is common [1]. A major challenge of remaining abstinent is resisting the urge to smoke when encountering stimuli or environments previously predictive of tobacco use, or brief exposure to the drug itself. Simple exposure to these triggers can lead to potent cravings and ultimately a resumption of smoking behaviour.

Learning to overcome cravings and remain resistant to relapse is crucial to achieving long-term abstinence. This process can be accurately modelled in animals via the extinction and reinstatement paradigm [2]. In this context, extinction of drug-seeking is an active learning process, producing a competing drug memory (response ≠ drug) that dominates over the previous drug-taking response [3].

It is now well established that, like long-term memory formation, the encoding of long-term extinction memories requires active gene transcription [4]. Increased gene transcription is necessary to produce neuroadaptive changes in protein expression at the level of the synapse [5]. Epigenetic mechanisms play a key role in experience-dependent changes in gene transcription, and are becoming increasingly implicated in drug-induced neuroplastic changes.

There are numerous epigenetic changes that have been investigated with respect to animal behaviour. Of these, histone acetylation is the most widely studied regarding both long-term memory formation and drug addiction. Through controlling accessibility of transcription enzymes to DNA, histone acetylation facilitates (via histone acetyltransferases; HATs) or silences (via histone deacetylases; HDACs) gene expression and any resultant changes which may be crucial to the persistence of memories across time [6].

Early evidence for the role of histone acetylation in the extinction of conditioned behaviour comes from Pavlovian conditioned fear in rats, where administration of an HDAC inhibitor prior to each extinction session enhances long-term extinction memory formation [7–9]. More recently, histone acetylation has been implicated in learning about Pavlovian context-drug associations. Both the acquisition and extinction of a conditioned place preference (CPP) for cocaine [10–12] and morphine [13, 14] are facilitated by HDAC inhibition, although these effects appear to be both dose and timing dependent [15, 16]. In contrast, HDAC inhibition impairs the acquisition of nicotine-induced CPP [17], suggesting that the role of histone acetylation in drug memory formation may vary according to drug type.

Extending the results of Pavlovian conditioning to an instrumental paradigm is particularly important in the study of drug addiction, as this type of procedure has higher face validity, more closely approximating drug taking behaviour in humans [18]. To this end, several recent studies have implicated histone acetylation in the rewarding properties of cocaine [19–22] and alcohol [23]. However, these effects often rely on pre-session treatment, suggesting an interaction between histone acetylation and the acute rewarding, or aversive [23], properties of the drugs, rather than any influence on the formation of drug-associated memories per se. The involvement of histone acetylation in the extinction of instrumentally conditioned drug memories has yet to be reported.

Therefore the present study aimed to specifically address the involvement of histone acetylation in the extinction of nicotine self-administration and the subsequent susceptibility to relapse. First, the HDAC inhibitor, sodium butyrate (NaB), was administered immediately after each extinction session to facilitate the encoding of extinction memories. An additional group received NaB six hours after the end of the session to determine if any observed effect was specifically due to memory consolidation, or rather occurred simply as a consequence of NaB exposure (Experiment 1). A cue-extinction procedure was then utilised to increase responding across extinction sessions, maximising the possibility of detecting treatment effects across this period when low rates of responding are typically observed (Experiment 2). Finally, the effect of NaB on extinction and reinstatement of sucrose-seeking was examined to determine if any detected effects were specific to nicotine-seeking, or are a property of reward-related learning more generally (Experiment 3).

## Materials and Methods

### Subjects

Male Sprague Dawley rats (n = 90; 175 g–200 g; approximately six weeks of age; Animal Resource Centre, WA, Australia) were housed four per cage on a 12 h reverse light/dark cycle (lights off at 7 a.m). Rats in all experiments received *ad libitum* access to food and water until two days prior to self-administration when food was restricted to 20 g/rat/day [24]. Rats were weighed weekly to ensure consistent weight gain across the experiment.

All procedures were approved by the Animal Care and Ethics Committee of The University of New South Wales (protocol number 12/155B) and the Macquarie University Animal Ethics Committee (protocol number 2011/007) and were conducted in accordance with the Australian Code for the Care and Use of Animals for Scientific Purposes (8^th^ ed, 2013). Surgeries were performed under isoflurane anaesthesia, and all efforts were made to minimise the number of animals used and their suffering.

### Drugs

Nicotine hydrogen tartrate and NaB (Sigma, St. Louis, MO, USA) were dissolved in sterile saline (0.9% NaCl). Nicotine solutions for subcutaneous (s.c.) priming injections were pH adjusted to 7.4 using sodium hydroxide. All doses of nicotine are reported as free base.

### Intravenous Nicotine Self-Administration

Two weeks after arrival, rats in Experiments 1 and 2 underwent surgery for the implantation of an intravenous catheter as described previously [25].

All self-administration studies were carried out in 8 standard self-administration chambers (Med Associates, VT, USA) housed within wooden sound attenuation boxes [25]. Chambers were equipped with a magazine, which separated two nose-poke holes assigned as ‘active’ or ‘inactive’ (counterbalanced left versus right).

Training began with two daily 1 hr habituation sessions with nose-pokes covered and house-light on. On the third day, nose-pokes were revealed and 12 daily 1 hr nicotine-self administration sessions commenced. Each response on the active nose-poke resulted in an infusion of nicotine (30 μg/kg/100 μL over 3 s), presentation of a cue-light within the nose-poke hole (3 s) and extinction of the house-light (20 s). Responses during the 20 s time-out or on the inactive nose-poke were recorded but were of no consequence. Rats were considered to have acquired self-administration if they achieved a minimum of six infusions/session across the last three sessions and showed a preference for the active nose-poke (2 active: 1 inactive).

### Experiment 1: The effect of HDAC inhibition on the extinction and reinstatement of nicotine-seeking behaviour

**Fig 1:**
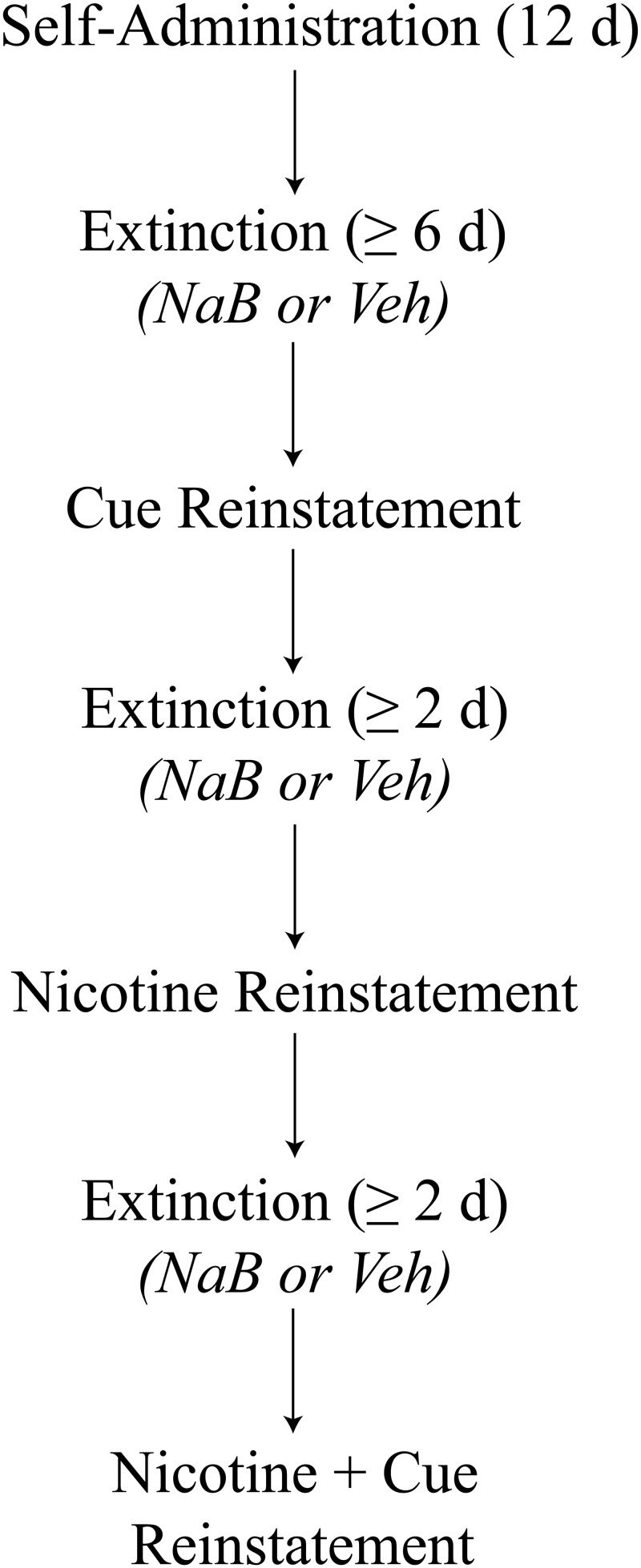
Schematic representation of the procedure of Experiment 1. Rats were trained to self-administer nicotine for 12 days followed by removal of both nicotine and associated cues to extinguish responding. Across a minimum of six extinction sessions, rats were treated with either the HDAC inhibitor, sodium butyrate (NaB; immediately or six hours following each session), or vehicle (Veh). Reinstatement of nicotine-seeking was then assessed through three reinstatement tests, each separated by a minimum of two further extinction sessions.

Forty two rats were used to examine the effect of NaB treatment on the extinction and reinstatement of nicotine-seeking. A schematic representation of the experimental design can be seen in Fig 1.

#### Extinction

Following the conclusion of self-administration training, daily extinction sessions commenced by removing nicotine and its associated cues. Based on performance during the last three days of acquisition (average active nose-pokes), rats were allocated to one of three treatment groups. Group ‘NaB’ and ‘NaB + 6 h’ received an intraperitoneal (i.p.) injection of the HDAC inhibitor, NaB (100 mg/kg at 1 ml/kg), immediately or six hours after each extinction session, respectively. This dose was selected as it has been shown to modulate cocaine-induced behavioural changes without affecting locomotor activity [26]. Group ‘Saline’ received an injection of saline (1 ml/kg) immediately after each session. Extinction continued for a minimum of six days or until the extinction criteria were met, defined here as a reduction in active responses to less than 30% (or a maximum of 6 responses) of the last three days of acquisition for two consecutive days [27]. Responses on both nose-pokes were recorded but were of no consequence.

#### Reinstatement

Once rats had achieved the extinction criteria, reinstatement was assessed during three tests each separated by a minimum of two re-extinction sessions. NaB or saline injections were again administered across re-extinction.

Testing began with a drug-free cue-reinstatement test where response-contingent cues were available throughout the session. This was followed by a nicotine-primed reinstatement test (0.3 mg/kg at 1 ml/kg, s.c. immediately prior to the session under extinction conditions) and then a combined nicotine + cue reinstatement test (nicotine prime plus response-contingent cue presentation). NaB was not administered following reinstatement tests.

### Experiment 2: The effect of HDAC inhibition on cue-extinction of nicotine self-administration

In Experiment 1 it was possible that the effect of NaB on responding across extinction was not detected due to low levels of nose-poking. Experiment 2 addressed this possibility with 24 additional rats that continued to receive the response-contingent cue across extinction, increasing responding across this phase.

#### Extinction and Reinstatement

Conditions during extinction were identical to those during initial self-administration, except that saline was substituted for nicotine (i.e. cues remained available in a response-contingent manner). Immediately following each extinction session, rats received an i.p injection of either NaB or saline. Once the extinction criteria were achieved or a maximum of 18 extinction sessions elapsed, a single nicotine + cue reinstatement test was conducted as described above.

### Experiment 3: The effect of HDAC inhibition on the extinction and reinstatement of sucrose-seeking

To determine whether the effects of NaB were specific to the extinction and reinstatement of drug-seeking, Experiment 3 examined the effect of NaB on the extinction and reinstatement of sucrose-seeking.

#### Acquisition

Parameters for sucrose self-administration were identical to those for nicotine with three exceptions: (1) rats did not undergo surgery; (2) active nose-pokes were reinforced with a 45 mg sucrose pellet (AIN-76A; TestDiet, MO, USA) delivered into the magazine; and (3) sessions lasted until a maximum of 30 pellets were earned or 30 minutes had elapsed.

#### Extinction and Reinstatement

Following acquisition, rats treated with either NaB or saline (immediately following each extinction session) underwent the same extinction/reinstatement procedure described in Experiment 1. Food-primed reinstatement tests involved placing 3 sucrose pellets in the magazine prior to test.

### Statistical Analysis

For all experiments, active and inactive nose-pokes across the last 3 days of acquisition were analysed using a mixed model ANOVA with the between-subjects factor of treatment (NaB, Saline [Expts. 2 and 3], and NaB + 6 h [Expt. 1]), and the within-subjects factor of nose-poke (active verses inactive).

Total active and inactive responses during the first six days of extinction were analysed using a mixed model ANOVA with the between-subjects factor of treatment and the within-subjects factors of session and nose-poke. As the greatest effect of NaB was expected on the first session after treatment [10], a planned contrast compared active and inactive nose-pokes amongst the treatment groups across the first two days of extinction. A Kaplan-Meier Survival analysis was also conducted to determine whether the number of extinction sessions required to reach criterion differed amongst groups in Experiment 2 [28].

Active and inactive responses during reinstatement were analysed using planned orthogonal contrasts, with the between-subjects factor of group, and the within subjects factor of nose-poke [29]. As responding declined rapidly across the session, analysis of these data were confined to the first 30 minutes of each test. In Experiments 2 and 3, responding in group NaB was compared to that in group Saline. In Experiment 1, the critical contrast examined whether responding in group NaB differed from that in the remaining groups (Saline and NaB + 6 h). The second contrast examined whether responding in group Saline differed from that in group NaB + 6 h. Alpha was fixed at 0.05 for all analyses.

## Results

### Experiment 1: NaB immediately, but not six hours, after extinction attenuates reinstatement of nicotine-seeking

Twelve rats were excluded from data analysis due to a failure to satisfy the acquisition criteria or loss of catheter patency (NaB = 5; Saline = 4; NaB + 6 h = 3). One rat (NaB) was excluded due to responding more than 2 standard deviations above the mean on reinstatement tests.

#### Acquisition

**Fig 2:**
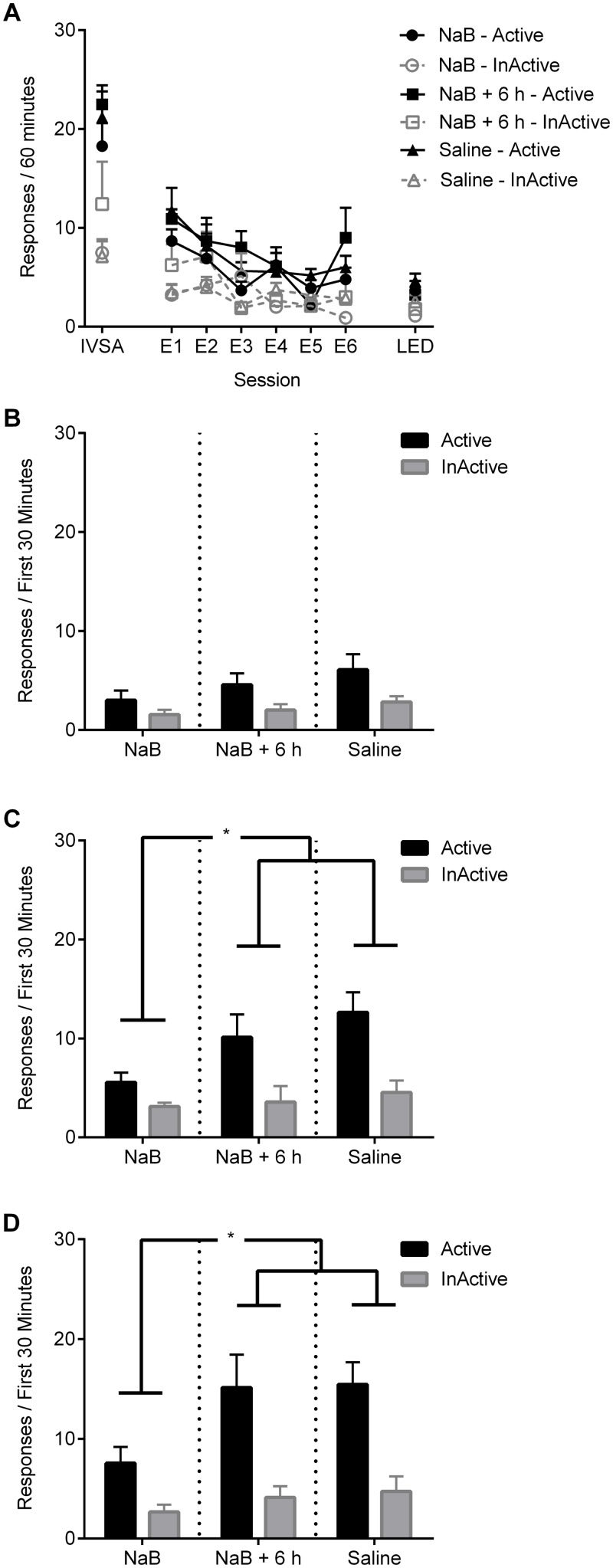
Experiment 1: NaB administered immediately, but not six hours, following extinction sessions attenuates reinstatement of nicotine-seeking. (A) Active and inactive responses during intravenous nicotine self-administration (IVSA; average of the last 3 sessions) as well as the first six days (E1–E6) and last day (LED) of extinction for rats treated with sodium butyrate (NaB; n = 9), saline (n = 11), or NaB administered six hours after the session (NaB + 6 h; n = 9). Bar graphs show active and inactive responses for each treatment group during the first 30 minutes of cue-reinstatement (B), nicotine-primed reinstatement (C) and nicotine + cue reinstatement (D). Data points and bars represent group means ± SEM. Asterisks indicate significant differences when p < 0.05.

At the conclusion of training rats clearly discriminated the drug source (main effect of nose-poke: F_1,26_ = 33.15, p < 0.001; Fig 2A). Treatment groups did not differ on baseline responding during the last three days of training (F<1).

#### Extinction

Following removal of nicotine and associated cues, responding on the active nose-poke declined across sessions (nose-poke x session interaction; F_5,22_ = 6.60, p < 0.01, Fig 2A). A significant session by treatment interaction (F_10,46_ = 2.12, p < 0.05), indicated that this reduction differed among treatment groups, however, this appears to be due to high levels of inactive responses in the NaB + 6 h condition only (Fig 2A). There were no group differences in the number of extinction sessions received, or in active and inactive responding on the days prior to the reinstatement tests (ps > 0.05).

#### Reinstatement

Responding across cue-reinstatement was greater on the active nose-poke (main effect of nose-poke: F_1,26_ = 10.15, p < 0.01, Fig 2B), yet was equivalent across treatment groups (Fs<1).

During nicotine-primed reinstatement, responding on the active nose-poke was significantly reduced in the NaB group compared to NaB + 6 h and saline groups (main effect of treatment: F_1,26_ = 4.26, p < 0.05; treatment x nose-poke interaction: F_1,26_ = 4.78, p < 0.05; Fig 2C). There were no differences in responding between saline and NaB + 6 h groups (Fs < 1), indicating that treatment with NaB six hours after the extinction session had no significant effect on nicotine-primed reinstatement.

The nicotine + cue reinstatement test produced a similar result (Fig 2D). Treatment with NaB immediately, but not six hours after each extinction session significantly blocked reinstatement of nicotine-seeking (main effect of treatment: F_1,25_ = 5.42, p < 0.05), and this difference was specific to responding on the active nose-poke (treatment x nose-poke interaction: F_1,25_ = 6.03, p < 0.05). Active and inactive responding were equivalent amongst groups Saline and NaB + 6 h (Fs<1).

Therefore, when administered immediately, but not six hours following extinction, NaB attenuated reinstatement by nicotine alone, or in combination with response-contingent cues.

### Experiment 2: HDAC inhibition facilitates cue-extinction of nicotine self-administration

Seven rats were excluded from data analysis due to a failure to satisfy the acquisition criteria or loss of catheter patency (NaB = 3; Saline = 4).

#### Acquisition

**Fig 3:**
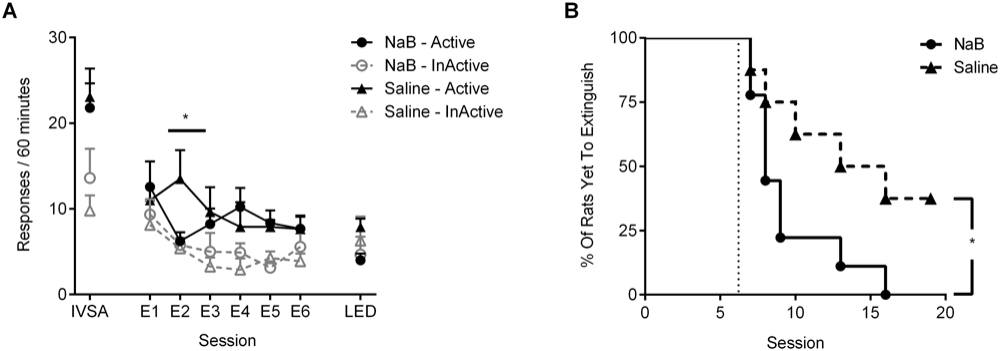
Experiment 2: HDAC inhibition facilitates cue-extinction of nicotine self-administration. (A) Active and inactive responses during acquisition of intravenous nicotine self-administration (IVSA; average of the last 3 sessions) and the first six days (E1–E6) and last day (LED) of extinction for rats treated with sodium butyrate (NaB; n = 9) and saline (n = 8) immediately after each extinction session; (B) Survival curve showing the percentage of rats in each group yet to meet the extinction criteria (active responses < 30% of last three days of acquisition). Dotted vertical line indicates the minimum number of extinction sessions.

Responding across the last 3 days of training was greater on the drug-paired nose-poke (main effect of nose-poke: F_1,15_ = 30.64, p < 0.001, Fig 3A). There were no differences in baseline self-administration between treatment groups.

#### Extinction

When cues were available to elevate responding across extinction an effect of NaB was now observed (session x group interaction: F_5,11_ = 3.74, p < 0.05, Fig 3A). The planned contrast revealed that the greatest difference between groups was on the first session after treatment (session x group interaction on day 1 vs day 2 of extinction: F_1,15_ = 5.55, p < 0.05).

Treatment with NaB also accelerated the rate of extinction, as treated rats required significantly fewer days to reach the extinction criteria (active responses less than 30% of baseline self-administration; χ^2^ = 4.39, df = 1, p < 0.05; Fig 3B). Indeed, 38% of rats in the saline condition failed to reach criteria at all (maximum 18 extinction sessions). This result indicates treatment with NaB markedly facilitates the extinction of nicotine-seeking that is otherwise maintained by previously drug-associated cues.

#### Reinstatement

Following extinction, responding during the nicotine-primed reinstatement test was equivalent across treatment groups (Fs<1, data not shown). However, it must be noted that the interpretation of this data is limited given the group differences in the number of extinction days received, and that several saline animals failed to extinguish at all prior to test.

### Experiment 3: HDAC inhibition has no effect on the extinction and reinstatement of sucrose-seeking

#### Acquisition

**Fig 4:**
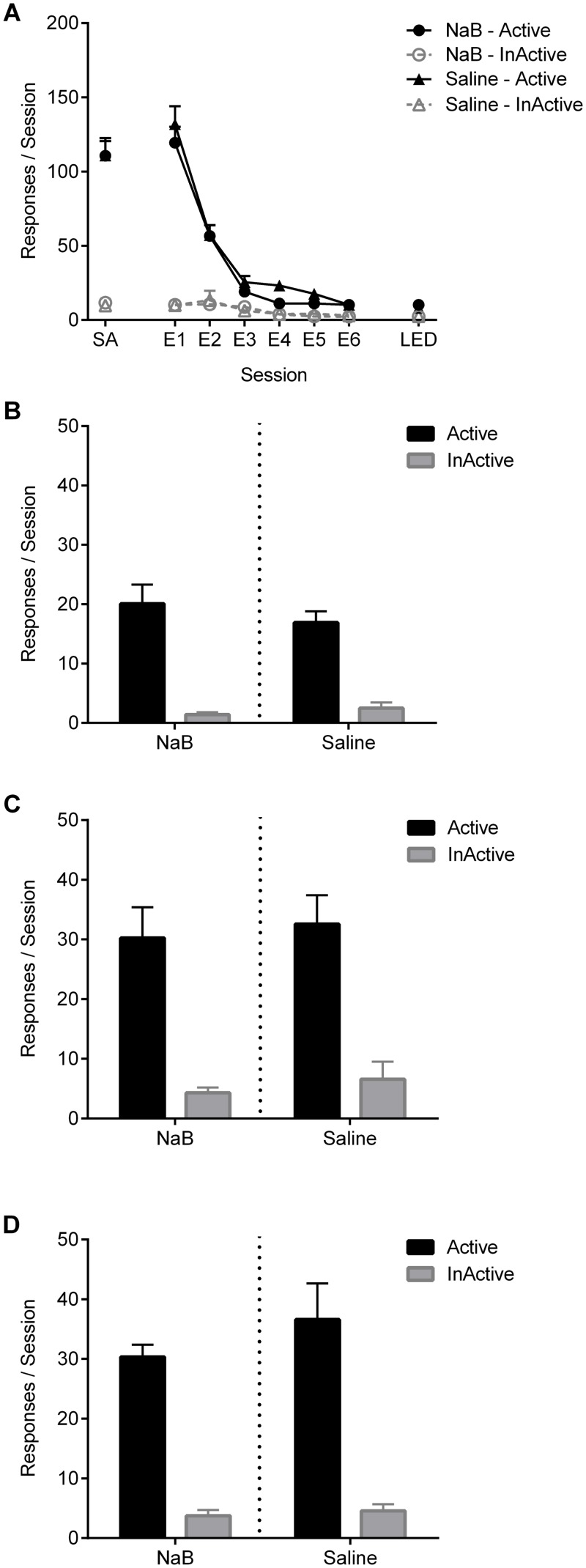
Experiment 3: HDAC inhibition has no effect on the extinction and reinstatement of sucrose-seeking. (A) Active and inactive responses during acquisition of sucrose self-administration (SA; average of the last 3 sessions) and the first six days (E1–E6) and last day (LED) of extinction for rats treated with sodium butyrate (NaB; n = 12) or saline (n = 12) immediately after each extinction session. Bar graphs show active and inactive responses for each treatment group during cue-reinstatement (B), food-primed reinstatement (C) and food + cue reinstatement (D). Data points and bars represent group means ± SEM. **Note:** during acquisition rats are removed from chambers if a maximum of 30 rewards are obtained.

All rats rapidly learned to nose-poke for sucrose pellets, resulting in a strong preference for the active nose-poke (main effect of nose-poke: F_1,22_ = 193.60, p < 0.001, Fig 4A). There were no differences between treatment groups across the last 3 days of training.

#### Extinction

Following removal of sucrose and associated cues, a significant reduction in responding on the active nose-poke was observed (nose-poke x session interaction: F_5,18_ = 32.82, p < 0.001, Fig 4A). Despite initial high levels of responding across extinction, no effect of NaB treatment was detected. Treatment groups did not differ on the amount of extinction sessions received, or on active and inactive responding on days prior to reinstatement tests.

#### Reinstatement

Across all three reinstatement tests, rats responded preferentially on the active nose-poke (main effect of nose-poke: F_1,22_ > 61.87, p < 0.001 for all; Fig 4B– 4D). No group differences were observed on any of the reinstatement tests (Fs < 1). Therefore, treatment with NaB across extinction of responding for sucrose had no effect on subsequent reinstatement of sucrose-seeking.

## Discussion

This present series of experiments show for the first time that HDAC inhibition facilitates the extinction of nicotine-seeking in a persistent manner that provides resistance to reinstatement. It is also the first demonstration that increasing histone acetylation during the period of extinction memory consolidation facilitates the extinction of an intravenously self-administered drug.

In Experiment 1, administration of NaB immediately following extinction sessions attenuated reinstatement of nicotine-seeking. This was evident as a marked decrease in active responding in the NaB treated rats during the nicotine alone and combined nicotine + cue reinstatement tests. Importantly, NaB did not impact inactive nose-poking, nor locomotor activity (S1 Fig). This indicates that the reduction in active responding was specific to the conditioned aspect of responding and not due to a general decrease in motivation, or differential sensitivity to the nicotine-prime. This finding is consistent with previous reports using CPP, where HDAC inhibition during extinction reduces reinstatement of a preference for a context previously paired with cocaine [10, 14, 15], and now extends these findings to extinction of responding for a drug reward using an instrumental conditioning paradigm.

The attenuation of reinstatement by NaB appears to be specific to the consolidation of extinction memories, as administration of NaB six hours after each extinction session had no impact on subsequent reinstatement of nicotine-seeking. The finding that the effect of NaB is temporally restricted supports previous studies suggesting that HDAC inhibition across extinction has no effect on cocaine-primed reinstatement when delivered outside the consolidation window [10]. It is also consistent with the hypothesis that long-term memory is dependent on a temporally-limited phase of transcription and protein synthesis [4].

Importantly, the absence of an effect in the rats treated six hours after the session also confirms that NaB treatment did not interfere with the rewarding properties of nicotine at test. This is distinct from past research showing that NaB reduces reinstatement of cocaine-seeking when administered prior to the reinstatement test session [20]. In the latter case, injection of NaB may be interfering with the acute rewarding properties of the drug [19–23], as separate from influencing the expression of drug-related memories.

Although NaB produced a robust decrease in responding during nicotine and nicotine + cue reinstatement, there was no effect detected on the cue-reinstatement test. The absence of an effect on cue-reinstatement may be attributable to the low levels of responding observed across this test session. Previously nicotine-associated cues have been shown to produce reliable reinstatement of responding [30], however, this appears to be sensitive to subtle variations in parameters, including training dose, response operandum, and repeated testing [31–33]. The use of parameters which elevate responding to nicotine-paired cues may allow for future experiments to determine whether the observed results may be extended to cue-reinstatement.

In Experiment 1, extinction of nicotine-seeking was very rapid, decreasing immediately following removal of nicotine and associated cues. Such low levels of responding may have obscured any evidence of the HDAC inhibitor-potentiated extinction learning that has been reported previously in CPP studies using other drugs of abuse [11]. To address this concern, a cue-extinction procedure was conducted in order to maintain responding across this phase [34]. Under these conditions, a facilitation of extinction learning by NaB was now clearly evident. Not only did NaB treatment reduce nose-poking on the first session following treatment (when response rates were greatest), but a marked reduction in the number of sessions required for rats to reach the extinction criteria was also detected. The observation that NaB facilitates learning about nicotine-paired discrete cues supports previous research demonstrating that HDAC inhibitors facilitate learning about Pavlovian conditioned stimuli [35]. The absence of an effect on reinstatement was not surprising, given that several saline rats failed to reach the extinction criteria, as responding did not decline among these animals on the days prior to test. Therefore, this study provides the first evidence that increasing histone acetylation across extinction both facilitates extinction learning and attenuates reinstatement of an intravenously self-administered drug reward.

The effect of HDAC inhibition appears to be specific to nicotine-associated memories, as NaB had no effect on the extinction or reinstatement of sucrose-seeking, despite the high levels of responding during these sessions. It also confirms that treatment with NaB was not exerting non-specific effects (i.e. mediated conditioning) on reward-related learning. This finding is consistent with previous research demonstrating that increasing histone acetylation decreases cocaine and ethanol, but not sucrose, self-administration in rats [19, 22, 23]. It may be the case that the altered acetylation state produced by nicotine [36], but not sucrose, results in a behaviour that is more amenable to manipulation by HDAC inhibitors, particularly at the lower NaB dose used here. Future studies may attempt to replicate this finding with other drugs of abuse, as exposure to stimulants such as cocaine also increases histone acetylation in brain reward circuitry [26].

The results of the present series of experiments support the growing body of research suggesting that HDACs are crucial negative regulators of long-term memory [37], and that their inhibition facilitates the extinction of drug-seeking. A remaining question from this study is what specific HDACs NaB is targeting to facilitate consolidation of extinction memories. Sodium butyrate is primarily an inhibitor of class I HDACs (HDAC1, 2, 3, 8). Both HDAC1 and 2 activity in the hippocampus have been heavily implicated in other learning and memory in paradigms, such as conditioned fear [38, 39]. Little research has investigated the role of specific HDACs in the extinction of drug-seeking. In one study, Malvaez and colleagues [11] demonstrated that administration of an HDAC3-selective inhibitor facilitates the extinction of cocaine-induced CPP, and is associated with immediate early gene expression in the nucleus accumbens, infralimbic cortex and hippocampus. Future studies using more specific compounds administered directly into brain will be able to elucidate the role of individual HDACs in the extinction of drug-seeking.

In summary, this study shows for the first time that treatment with the HDAC inhibitor, NaB, facilitates the extinction of nicotine-seeking in a manner that provides resistance to reinstatement. It is also the first study demonstrating that increasing histone acetylation during memory consolidation facilitates the extinction of an intravenously self-administered drug, a finding not observed when rats respond for a natural reward. Results of the present series of experiments provide further support for the role of HDACs as negative regulators of the extinction of drug-associated memories.

## Supporting Information

S1 Fig NaB has no effect on locomotor activity during extinction. Locomotor activity during acquisition of self-administration (IVSA/SA) and the first six days of extinction (E1–E6) for rats treated with sodium butyrate (NaB) or saline in (A) Experiment 1, (B) Experiment 2, and (C) Experiment 3. **Note**: SA sessions in Experiment 3 lasted until a maximum of 30 pellets were earned or 30 minutes had elapsed.
(TIF)
