# Supplementary figures and images for: Inhibition of Histone Deacetylases Facilitates Extinction and Attenuates Reinstatement of Nicotine Self-Administration in Rats
Source: PLoS One. 2015 Apr 16;10(4):e0124796. doi: 10.1371/journal.pone.0124796 (PMC4399837; doi:10.1371/journal.pone.0124796)

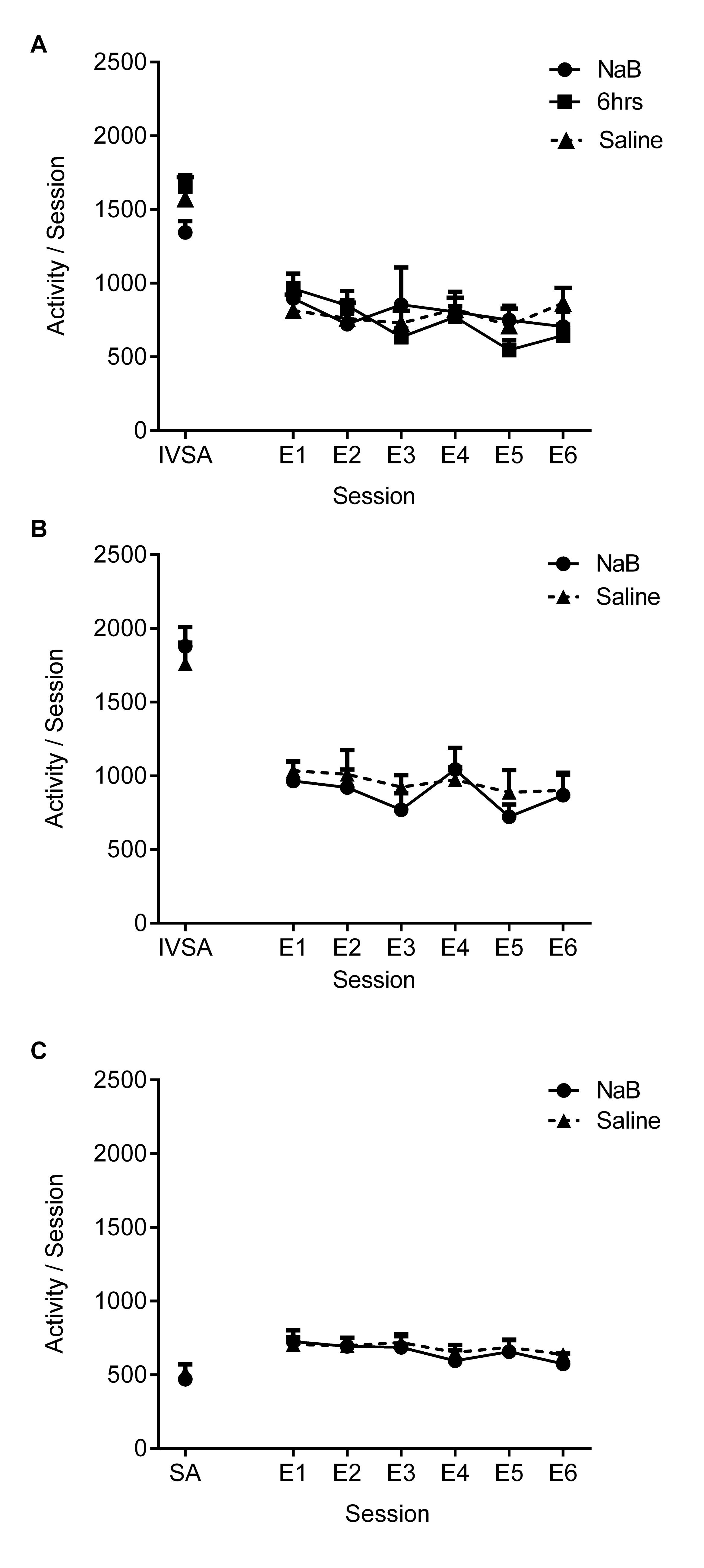

Supplement: S1 Fig — Locomotor activity during acquisition of self-administration (IVSA/SA) and the first six days of extinction (E1–E6) for rats treated with sodium butyrate (NaB) or saline in (A) Experiment 1, (B) Experiment 2, and (C) Experiment 3. Note: SA sessions in Experiment 3 lasted until a maximum of 30 pellets were earned or 30 minutes had elapsed. (TIF) [file pone.0124796.s001.tif]
